# Oral Tolerance Induction—Opportunities and Mechanisms

**DOI:** 10.3390/foods11213386

**Published:** 2022-10-27

**Authors:** Ru-Xin Foong, Alexandra F. Santos

**Affiliations:** 1 Department of Women and Children’s Health (Paediatric Allergy), School of Life Course Sciences, Faculty of Life Sciences and Medicine, King’s College London, London SE1 9RT, UK; 2 Children’s Allergy Service, Guy’s and St. Thomas’ NHS Foundation Trust, London SE1 7EH, UK; 3 Peter Gorer Department of Immunobiology, School of Immunology and Microbial Sciences, King’s College London, London SE1 9RT, UK; 4 Asthma UK Centre for Allergic Mechanisms of Asthma, London E1 8AA, UK

**Keywords:** food allergy, allergy diagnostic testing, allergy resolution

## Abstract

Oral tolerance is the active absence of response to food allergens, which involves complex mechanisms in the gut-associated lymphoid tissue. Food allergy results from the disruption of such tolerance or the absence of its establishment in the first place. It follows allergic sensitization with the production of allergen-specific IgE and results from the degranulation of basophils and mast cells on subsequent exposure to the allergen. Oral tolerance induction has been explored in the contexts of prevention and treatment of food allergy. Early introduction of allergenic foods (i.e., egg and peanut) in the diet of infants, before allergic sensitization occurs (i.e., via inflamed skin affected with eczema) has shown to be beneficial. Guidelines have changed to recommend the introduction of these allergenic foods by 6 months of age. For food allergic individuals, oral tolerance induction has been attempted using allergen-specific immunotherapy, which involves the administration of an allergen, modified or not, through various possible routes, including oral, sublingual, epicutaneous, and subcutaneous, with or without concomitant administration of antibody-based biologics. Further research into the immune mechanisms of food allergy and oral tolerance can lead to the identification of novel targets to suppress the food allergic response and reverse the current food allergy epidemic.

## 1. Introduction

Food allergy is defined as an abnormal immune response to a specific food. This can either occur due to a loss of oral tolerance or an inability to establish tolerance in the first instance. The prevalence and severity of food allergy is increasing on a global scale. With the absence of definitive treatment, most food allergic patients are advised to avoid the foods that cause their allergic reactions and must always carry emergency medications with them. Therefore, in recent years the search for new potential targets and the development of prevention strategies to reduce the food allergy epidemic has become the forefront of food allergy research.

## 2. Oral Tolerance Induction

The term “oral tolerance” is used to describe the physiological process whereby a person’s digestive system absorbs foreign food proteins after ingestion without causing a harmful immune response [1]. This process involves the gastrointestinal barrier, humoral factors, and various immune cell mechanisms that results in an immune response that ignores foreign antigens. The development of oral tolerance usually occurs during the infant weaning process when solid foods are introduced into their diets; although there is likely to be some initial exposure to food proteins via breastmilk and/or formula feeds (i.e., cow’s milk protein). Breastfeeding contributes to the establishment of an infant’s faecal microbiota including the colonization of the gut with Bifidobacteria. Bifidobacteria is thought to help promote mucosal tolerance through its interaction with regulatory T-lymphocytes and Toll-like receptors [2].

Although the most obvious route of food allergen exposure is through gut ingestion of the food, allergen exposure can also occur through the skin and respiratory tract. Several hypotheses exist as to how food allergies develop as some children’s reaction occurs on first oral exposure to the culprit food, which suggests sensitization to the food must somehow occur beforehand. One of these hypotheses is known as the dual-allergen exposure hypothesis, which suggests that the risk of developing a food allergy depends on the timing, dose, route of exposure, and the balance between oral exposure, which is tolerogenic, and cutaneous (or respiratory) exposure, which can be allergenic [3]. 

Allergic sensitization to foods can occur through a weakened skin barrier (i.e., the skin of children with eczema/atopic dermatitis) that allow allergens to enter the body and induce IgE-synthesis. Research performed on loss-of-function variants in the filaggrin gene (FLG) have been important in supporting this hypothesis. FLG is only expressed in the skin and is known to play an important role in epithelial barrier function and the development of eczema. Evidence has shown that children who carry an FLG mutation have an increased risk of peanut sensitization and, through early environmental peanut exposure, an increased risk of peanut allergy [4]. This is further supported by the Isle of Wight cohort where children with FLG mutations were at significantly greater risk of food allergy later in childhood [5]. It has been suggested that an impaired skin function barrier facilitates the uptake of epicutaneous antigens and causes an antibody-mediated response resulting in allergic sensitization, rather than exposure via the gut. If this initial allergic sensitization has already occurred, when an infant begins weaning and is exposed to the food orally, an IgE-mediated allergic reaction can happen. For those children where allergic sensitization does not occur via the skin, their first exposure to the food allergens in the gastro-intestinal tract allows for the establishment of oral tolerance. 

## 3. Immune Mechanisms Underlying Oral Tolerance

The underlying mechanism that contributes to the process of oral tolerance is linked to gut-associated lymphoid tissue (GALT). GALT is the largest immune organ in the body, whose role is to protect the body against pathogens and allow for the ingestion of dietary antigens in a way that does not cause immune reactions [6]. Immune tolerance can be defined as the antigen-specific suppression of cellular immune responses and this process is the default immune pathway in the gastrointestinal tract [7]. 

The gastrointestinal tract comprises the largest interface between the body and the external environment. It encounters a huge range of diverse foreign antigens, including over 30 kg of food proteins a year [8]. When food is ingested, food proteins are denatured and degraded into small peptides, which are absorbed and transported through various complex processes via antigen-presenting cells (APC) in the mucosal layer and then the intestinal epithelial cell barrier within the gut. This can occur through uptake via columnar epithelial cells, entering via M cells within Peyer’s patches (macroscopic lymphoid aggregates in the small intestine and mesenteric lymph nodes) or by dendritic cell processes that penetrate the gut lumen [6]. Dendritic cells (DC) in the gut that express the integrin CD11b and chemokine receptor CX3CR1 can also directly sample luminal content by forming transepithelial dendrites. Once the antigen is in the lamina propria layer of the gut, DC that express the integrin CD103 and CD11c promote the production of IgA and the differentiation of naïve T-cells into regulatory T (Treg) cells. Following this generation in the gut mesenteric lymph nodes, the Treg cells up-regulate CCR9 and integrin α4β7 to go back into the lamina propria where they undergo local expansion to induce tolerance. The Treg cells are, therefore, important in the promotion of B-cell class switching to produce non-inflammatory IgA responses, T-cell anergy of effector cells and inhibiting downstream pro-inflammatory cells, such as eosinophils, basophils, and mast cells, resulting in oral tolerance [9]. More specifically in the gut, Foxp3+ and IL-10 producing Foxp3-peripheral Tregs (pTregs) are an important subtype of Tregs in their ability to produce immunoregulatory cytokines, such as transforming growth factor beta (TGF-β) and IL-10, that contribute to these Treg cell functions. Although Treg-cells specific to food allergens are formed and mostly remain in the intestine, they can also be found in the circulation, which helps to maintain systemic tolerance [9]. 

## 4. Food Allergy—What Went Wrong?

Immune tolerance is driven by DC-mediated antigen presentation in the gut and represents a healthy response to food antigen exposure. When tissue is injured or inflamed, epithelial cells produce T-helper 2 (Th2) inducing cytokines, such as IL-25 and IL-33, which then act on different cells in the Th2 response including DC, mast cells, basophils, and innate lymphoid cells. In mouse models, IL-33 has been shown to have an important role in Th2 differentiation of naïve T cells, which are important cells that drive and perpetuate allergic responses [10]. Th2 cells are polarized to the production of IL-4, IL-13, IL-5, and IL-9, and it is these cytokines that can affect mucous secretion, eosinophil recruitment, and mast cell activation. If allergen-specific IgE is available and binds to the F_C_εRI receptors on the surface of mast cells and basophils, this leads to degranulation resulting in an allergic response. Although the exact mechanism for how oral tolerance is disrupted in people with food allergies is unknown, there are studies that show that a weakened ability to regenerate pTreg cells after exposure to a food allergen may be a factor that contributes to loss of tolerance [9]. Genetic predisposition factors, especially for increased skin and gut permeability, may also contribute to an increased risk of sensitization and, therefore, allergies. These predisposing factors include disruption to the skin barrier integrity in children with loss of function filaggrin mutations [5] and inflammatory skin diseases, such as atopic dermatitis [8]. 

Allergic sensitization starts when an allergen is captured by APC. These APC cells bind, process, and then present the allergen to naïve T-cell lymphocytes, which results in an antibody-mediated response from interactions with B lymphocytes. When the naïve allergen-specific Th2 cells bind to the allergen, clonal expansion of the cells occurs causing the release of Th-2 specific cytokines, such as interleukin-13 (IL13) and interleukin-4 (IL4). This, in turn, causes the production of B cells that express specific anti-IgE molecules on their surface for that allergen as a part of the B-cell receptor [11]. T follicular helper (Tfh) cells are critical in inducing B cell activation and differentiation. A new subset of Tfh has been described and designated Tfh13—it is characterized by IL-4 and IL-13 production and is able to induce B cells to produce high affinity IgE and to cause anaphylaxis following allergen exposure [12]. This is in contrast with Tfh2 cells, which also produce IL-4 and can induce IgE production, but of lower affinity. The functional characteristics modulate the ability of IgE to induce effector cell activation following allergen exposure—this has recently been demonstrated using a human peanut allergy in vitro model [13]. Class-switching to IgE and IgE production can also occur locally in the gastro-intestinal tissue [14]. When a sensitized individual is re-exposed to the same allergen, the IgE B-cells proliferate to IgE-secreting plasma cells with IgE antibodies remaining in the peripheral circulation. FcεRI receptors on mast cells and basophils bind to these IgE antibodies, initiating a rapid acute phase response leading to degranulation of pre-stored inflammatory mediators, such as histamine, heparin, and Th2 cytokines (IL-4, IL-5, IL-13). The release of these mediators causes local tissue responses in multiple organ systems, such as skin, respiratory tract, cardiovascular, gastrointestinal, neurological, or systemically, can cause anaphylaxis to occur [15]. 

For the patients who have been sensitized and then are exposed to an allergen, the cross-linking of allergen-specific IgE bound to FcεRI receptors on mast cells and basophils results in the release of proinflammatory mediators that are responsible for the immediate phase of an allergic reaction. A late phase reaction occurs due to the accumulation of inflammatory mediators produced by these effector cells and the activation of allergen-specific Th2 cells, which produce interleukins, promotes eosinophilia, maintains allergen-specific IgE levels, and recruits additional inflammatory cells to tissues resulting in worse tissue damage and inflammation [16]. The mechanisms involved in food allergic reactions have, therefore, become potential therapeutic targets when looking at food tolerance. 

## 5. Oral Tolerance Induction for Prevention of Food Allergy

**Figure 1:**
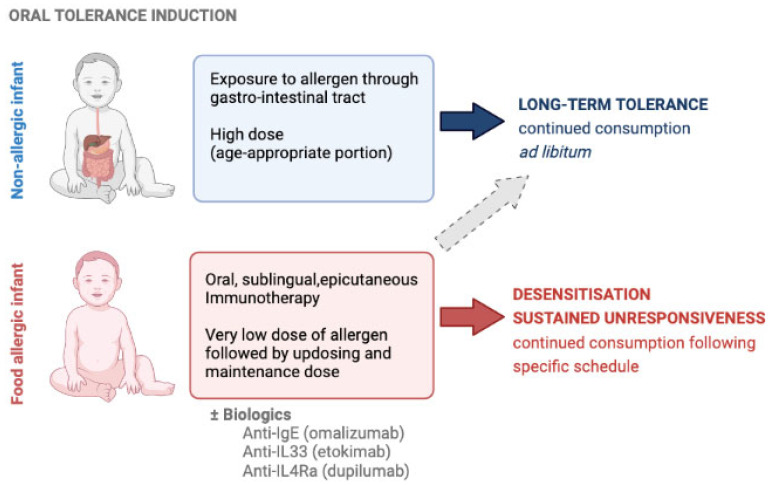
Oral tolerance induction can be achieved by timely introduction of the food in the infant’s diet and can be attempted in food allergic children using allergen-specific immunotherapy, possibly in conjunction with biologics, but these are subject of ongoing research.

The concept of oral tolerance induction for common allergens, such as egg, milk, and peanut, has been at the forefront of food allergy research over the last decade in the search for strategies to prevent the development of food allergy (Figure 1). Significant differences in the prevalence of peanut allergy in infants in different countries and its possible association with consumption of peanut earlier and in larger quantities having a protective effect on the development of peanut allergy in later childhood, as proposed in the dual-allergen exposure hypothesis, led to the design of the Learning Early About Peanut (LEAP) landmark study [17,18]. This randomized control trial (RCT) initiated an intervention where high-risk infants aged 4–11 months consumed an average of 6 g of peanut protein a week until 60 months of age. The prevalence of peanut allergy was assessed at 60 months of age and compared to the control group who were high-risk infants who were advised to avoid peanut entirely. The findings showed that early introduction and regular on-going consumption of peanut resulted in an 81% relative risk reduction in the number of children with peanut allergy compared to those who avoided peanut [18]. Another RCT, called the Enquiring About Tolerance (EAT) study, looked at early introduction of six allergenic foods (i.e., egg, cow’s milk, peanut, sesame, fish, and wheat) into the diets of breastfed infants at 3–4 months of age compared to breastfed infants who were advised to comply with normal weaning guidelines (i.e., introduce solids at 6 months). Their per protocol analysis showed that infants who introduced egg and peanut early (i.e., at 3–4 months of age) and consumed it regularly in high quantities were less likely to have these food allergies at 72 months of age [19]. Other oral tolerance induction studies have found similar findings. The Australian Healthnuts cohort found that a delayed introduction of egg until 10–12 months or after 12 months were both associated with a significantly increased risk of developing an egg allergy compared to those who introduced it early (i.e., 4–6 months of age) [20]. The PETIT RCT study also showed a statistically significant decrease in egg allergy in infants with eczema who had egg introduction early via a two-step process compared to those who received a placebo [21]. 

From these studies, there has been a change in the recommendations given with regards to early introduction of food allergens into infant’s diets. Ierodiakonou et al. conducted a systematic review on the timing of allergenic food introduction during infancy and reported moderate certainty evidence from two trials that early peanut introduction was associated with a reduction in peanut allergy [22]. The National Institute of Allergy and Infectious Diseases (NIAID) have changed their guidance on the introduction of peanut into infant diets and now recommend that infants with severe eczema, egg allergy, or both should have age-appropriate peanut-containing foods introduced into their diets as early as 4–6 months of age [23]. The European guidelines recommend the introduction of egg and peanut in the diet of infants, particularly in countries where these foods are endemically consumed [24]. The Earlynuts study from Australia has demonstrated a wide acceptance and integration of these new recommendations by the population with less than 3/10 infants consuming peanut before and more than 9/10 infants consuming peanut by the age of 12 months after the recommendations regarding introduction of peanut in the infants’ diet were issued [25]. This reflects the impact of the above studies and the change in practice that it has resulted in in some parts of the world.

## 6. Food Allergy and Possible Oral Tolerance Induction through Immunomodulatory Treatments

Interest in food allergens being used as potential immunomodulators has driven research into different disease-modifying therapeutic approaches using allergen in immunotherapy studies. Oral immunotherapy was first reported in 1908 by Schofield who successfully desensitized a child to egg by incorporating it into their diet [26]. Food oral immunotherapy (OIT) involves giving doses of a food allergen to the patient over three phases: the first is the initial rapid up-dosing, followed by an escalation phase, and then maintenance phase [27]. Desensitization rates have been variable depending on the food type, but also dosing of treatment. OIT for peanut has been one of the more well-studied foods. A recent OIT trial using peanut product AR101 found that with a 6-month period of escalation to a 300 mg dose of peanut protein followed by a 6-month period of maintenance dose at this level, 50% of participants could consume the equivalent of three peanuts after 1 year of treatment [28]. Another RCT (POISED), which is currently in the phase II stage, had patients building up to a 4 g peanut protein dose (equivalent to 16 peanuts) and then randomizing them to either reduce their dose or completely avoid peanuts [29]. Their results showed a decay curve in that if the participants avoided peanut for a year, they lost approximately 50% of their tolerance every 3 months over the course of a year. A slope of decay was also seen for those who reduced their dose to 300 mg, albeit at a slower rate. Although there is good evidence to suggest that OIT is efficacious, there is no clear evidence on how long OIT must be initiated for to allow for a degree of sustained responsiveness or, even better, permanent tolerance to food allergens. Various biomarkers have been studied to see whether they can help predict tolerance. One of the early immunological changes seen in OIT is an increase in food-specific IgG4 levels, which supports its use as a predictor of clinical outcome during food allergy OIT [16]. However, studies looking at immunotherapy, albeit for allergic rhinitis, have also reported that although sIgG4 is increased in active treatment groups, levels revert to pre-treatment levels if the treatment is stopped [16,30]. 

Other studies have focused on sublingual or epicutaneous administration of allergen-specific immunotherapy rather than oral. The PEPITES RCT looked at the effect of epicutaneous peanut immunotherapy (EPIT) compared to placebo on peanut protein ingestion among children with peanut allergy [31]. Their main outcome of measuring treatment effect was the response rate difference between the active and placebo treatment groups. Although there was a statistically significant difference found in the proportion of responders to EPIT in terms of desensitizing peanut allergic children to peanut protein, it did not meet their predicted 15% prespecified criterion to determine a positive trial outcome. However, from a sustained tolerance and safety point of view, they followed up patients who continued on EPIT for an additional 2 years (i.e., the PEOPLE study) and demonstrated that daily EPIT treatment for peanut allergy was a well-tolerated daily treatment [32]. Different formulations with modified allergens are able to prevent recognition by IgE and the release of mediators during an allergic reaction, which reduces side-effects, improves safety, and maintains immunogenicity, i.e., recognition by T-cell and subsequent responses. For instance, DNA peptide vaccines use DNA codes for the major peanut allergens, Ara h 1, Ara h 2, Ara h 3, and additional sequence encoding lysosomal associated membrane protein (ClinicalTrials.gov Identifier: NCT03755713). Another example being tested are viral-like particles containing the major peanut allergen Ara h 2, which have shown to be safe and effective in mouse models and suppress the Th2 immune response in human in vitro models [33].

Antibody-based treatments, including passive immunization, have been studied in food allergy research and have shown promise in early clinical studies of respiratory allergy. In mouse models, passive immunization with allergen-specific IgG antibodies in mice sensitized to birch pollen showed an 80% reduction of allergen-specific IgE binding and basophil degranulation [34]. In a recently published phase I study, an antibody targeting Bet v 1 was shown to be safe and reduced the symptoms in patients with birch pollen allergy [35]. There has also been increasing interest in the role of anti-IgE biologics to treat food allergies. Omalizumab is a recombinant DNA-derived humanized monoclonal antibody that binds to human IgE, which results in a significant decrease in free IgE in serum preventing it from binding to effector cells. This, in turn, means decreased mediator release and down-regulation of the FcεRI receptors on mast cells and basophils [36], reducing the risk of an allergic reaction. Omalizumab has been used in combination with food immunotherapy in a few small pilot and double-blind placebo-controlled trials for milk and peanut, and there is some evidence to show that the addition of omalizumab to OIT can decrease the time required to reach the maintenance OIT dose and reduces adverse effects [36]. Research around other biologics is becoming of increasing interest in their ability to inhibit the release of specific Th2 cytokines. In a pilot phase 2a study, participants were given a single dose of etokimab (anti-IL-33) to desensitize peanut allergic adults. Although the study was small (*n* = 20), a single dose of etokimab was safe, well tolerated, and those participants that received the dose showed a significant increase in desensitization to peanut protein [37]. Other agents, such as dupilumab (anti-interleukin 4Ra and anti-interleukin 13), have shown promising results in reducing other atopic conditions, such as eczema and eosinophilic asthma, so the potential for future use in desensitizing food allergic patients is plausible.

So far, the efficacy of immunomodulatory treatments has been variable, with actual oral tolerance induction achieved in only a small proportion of allergic patients and side-effects hampering further development or patients’ adherence to existing options. It remains to be proven whether immunotherapy can lead to long-term oral tolerance, where individuals may consume the food at any frequency in any amount without developing any allergic symptoms. A greater understanding of the immune mechanisms involved in food allergy and oral tolerance may lead us to a better efficacy and safety profile for future disease-modifying therapeutic approaches (Figure 1). 

## 7. Conclusions

Early introduction of allergenic foods in the diet of infants has shown to be tolerogenic and beneficial in infants that have not yet developed food allergy. Measures targeting other routes of sensitization, that are prone to allergic sensitization, are likely to complement oral tolerance induction in the prevention of food allergy. Oral tolerance involves numerous complex immunological processes that enable an individual to tolerate potential allergens. The understanding of these processes continues to evolve, and has paved the way to better understanding potential preventive and therapeutic interventions, including allergen-specific immunotherapy and use of biologics in the treatment of food allergy. Further research into the mechanisms of oral tolerance, but also the optimal dosing and timing at which tolerance develops naturally, are areas for future development, which will aid in the identification of new targets for effective prevention and treatment of food allergies to help reverse the current food allergy epidemic.

## Data Availability

Data is contained within the article.

## References

[1] Matsumoto K., Saito H. (2013). Epicutaneous Immunity and Onset of Allergic Diseases-Per-“Eczema”tous Sensitization Drives the Allergy March. Allergol. Int..

[2] Heine R.G. (2018). Food Allergy Prevention and Treatment by Targeted Nutrition. Ann. Nutr. Metab..

[3] Du Toit G., Foong R.M., Lack G. (2016). Prevention of food allergy—Early dietary interventions. Allergol. Int..

[4] Brough H.A., Simpson A., Makinson K., Hankinson J., Brown S., Douiri A., Belgrave D.C., Penagos M., Stephens A.C., McLean W.I. (2014). Peanut allergy: Effect of environmental peanut exposure in children with filaggrin loss-of-function mutations. J. Allergy Clin. Immunol..

[5] Venkataraman D., Soto-Ramírez N., Kurukulaaratchy R.J., Holloway J.W., Karmaus W., Ewart S.L., Arshad S.H., Erlewyn-Lajeunesse M. (2014). Filaggrin loss-of-function mutations are associated with food allergy in childhood and adolescence. J. Allergy Clin. Immunol..

[6] Faria A.M., Weiner H.L. (2005). Oral tolerance. Immunol Rev..

[7] Vickery B.P., Scurlock A.M., Jones S.M., Burks A.W. (2011). Mechanisms of immune tolerance relevant to food allergy. J. Allergy Clin. Immunol..

[8] Chinthrajah R.S., Hernandez J.D., Boyd S.D., Galli S.J., Nadeau K.C. (2016). Molecular and cellular mechanisms of food allergy and food tolerance. J. Allergy Clin. Immunol..

[9] Wambre E., Jeong D. (2017). Oral Tolerance Development and Maintenance. Immunol. Allergy Clin. North Am..

[10] Schoos A.-M.M., Bullens D., Chawes B.L., Costa J., De Vlieger L., DunnGalvin A., Epstein M.M., Garssen J., Hilger C., Knipping K. (2020). Immunological Outcomes of Allergen-Specific Immunotherapy in Food Allergy. Front. Immunol..

[11] Anvari S., Miller J., Yeh C.Y., Davis C.M. (2019). IgE-Mediated Food Allergy. Clin. Rev. Allergy Immunol..

[12] Gowthaman U., Chen J.S., Zhang B., Flynn W.F., Lu Y., Song W., Joseph J., Gertie J.A., Xu L., Collet M.A. (2019). Identification of a T follicular helper cell subset that drives anaphylactic IgE. Science.

[13] Hemmings O., Niazi U., Kwok M., James L.K., Lack G., Santos A.F. (2021). Peanut diversity and specific activity are the dominant IgE characteristics for effector cell activation in children. J. Allergy Clin. Immunol..

[14] Croote D., Darmanis S., Nadeau K.C., Quake S.R. (2018). High-affinity allergen-specific human antibodies cloned from single IgE B cell transcriptomes. Science.

[15] De Martinis M., Sirufo M.M., Suppa M., Ginaldi L. (2020). New Perspectives in Food Allergy. Int. J. Mol. Sci..

[16] Sindher S.B., Long A., Acharya S., Sampath V., Nadeau K.C. (2018). The Use of Biomarkers to Predict Aero-Allergen and Food Immunotherapy Responses. Clin. Rev. Allergy Immunol..

[17] Du Toit G., Katz Y., Sasieni P., Mesher D., Maleki S.J., Fisher H.R., Fox A.T., Turcanu V., Amir T., Zadik-Mnuhin G. (2008). Early consumption of peanuts in infancy is associated with a low prevalence of peanut allergy. J. Allergy Clin. Immunol..

[18] Du Toit G., Roberts G., Sayre P.H., Bahnson H.T., Radulovic S., Santos A.F., Brough H.A., Phippard D., Basting M., Feeney M. (2015). Randomized trial of peanut consumption in infants at risk for peanut allergy. N. Engl. J. Med..

[19] Perkin M.R., Logan K., Tseng A., Raji B., Ayis S., Peacock J., Brough H., Marrs T., Radulovic S., Craven J. (2016). Randomized Trial of Introduction of Allergenic Foods in Breast-Fed Infants. N. Engl. J. Med..

[20] Allen K.J., Koplin J.J., Ponsonby A.-L., Gurrin L.C., Wake M., Vuillermin P., Martin P., Matheson M., Lowe A., Robinson M. (2013). Vitamin D insufficiency is associated with challenge-proven food allergy in infants. J. Allergy Clin. Immunol..

[21] Natsume O., Kabashima S., Nakazato J., Yamamoto-Hanada K., Narita M., Kondo M., Saito M., Kishino A., Takimoto T., Inoue E. (2016). Two-step egg introduction for prevention of egg allergy in high-risk infants with eczema (PETIT): A randomised, double-blind, placebo-controlled trial. Lancet.

[22] Caffarelli C., Garrubba M., Greco C., Mastrorilli C., Povesi Dascola C. (2016). Asthma and Food Allergy in Children: Is There a Connection or Interaction?. Front. Pediatr..

[23] Togias A., Cooper S.F., Acebal M.L., Assa'ad A., Baker J.R., Beck L.A., Block J., Byrd-Bredbenner C., Chan E.S., Eichenfield L.F. (2017). Addendum Guidelines for the Prevention of Peanut Allergy in the United States: Report of the National Institute of Allergy and Infectious Diseases-Sponsored Expert Panel. Pediatr. Dermatol..

[24] Halken S., Muraro A., de Silva D., Khaleva E., Angier E., Arasi S., Arshad H., Bahnson H.T., Beyer K., Boyle R. (2021). EAACI guideline: Preventing the development of food allergy in infants and young children (2020 update). Pediatr. Allergy Immunol..

[25] Soriano V.X., Peters R.L., Ponsonby A.L., Dharmage S.C., Perrett K.P., Field M.J., Knox A., Tey D., Odoi S., Gell G. (2019). Earlier ingestion of peanut after changes to infant feeding guidelines: The EarlyNuts study. J. Allergy Clin. Immunol..

[26] Schofield A. (1908). A Case of Egg Poisoning. Lancet.

[27] Ramesh M., Karagic M. (2018). New modalities of allergen immunotherapy. Hum. Vaccines Immunother..

[28] Hogan S., Vickery B.P., Vereda A., Casale T.B., Beyer K., du Toit G., Hourihane J.O., Jones S.M., Shreffler W.G., PALISADE Group of Clinical Investigators (2020). Faculty Opinions recommendation of AR101 oral immunotherapy for peanut allergy. N. Engl. J. Med..

[29] Chinthrajah R.S., Purington N., Andorf S., Long A., O'Laughlin K.L., Lyu S.C., Manohar M., Boyd S.D., Tibshirani R., Maecker H. (2019). Sustained outcomes in oral immunotherapy for peanut allergy (POISED study): A large, randomised, double-blind, placebo-controlled, phase 2 study. Lancet.

[30] Shamji M.H., Kappen J.H., Akdis M., Jensen-Jarolim E., Knol E.F., Kleine-Tebbe J., Bohle B., Chaker A.M., Till S.J., Valenta R. (2017). Biomarkers for monitoring clinical efficacy of allergen immunotherapy for allergic rhinoconjunctivitis and allergic asthma: An EAACI Position Paper. Allergy.

[31] Fleischer D.M., Greenhawt M., Sussman G., Begin P., Nowak-Wegrzyn A., Petroni D., Beyer K., Brown-Whitehorn T., Hebert J., O'B Hourihane J. (2019). Effect of Epicutaneous Immunotherapy vs Placebo on Reaction to Peanut Protein Ingestion Among Children With Peanut Allergy: The PEPITES Randomized Clinical Trial. JAMA.

[32] Fleischer D.M., Shreffler W.G., Campbell D.E., Green T.D., Anvari S., Assa’Ad A., Bégin P., Beyer K., Bird J.A., Brown-Whitehorn T. (2020). Long-term, open-label extension study of the efficacy and safety of epicutaneous immunotherapy for peanut allergy in children: PEOPLE 3-year results. J. Allergy Clin. Immunol..

[33] Storni F., Zeltins A., Balke I., Heath M.D., Kramer M.F., Skinner M.A., Zha L., Roesti E., Engeroff P., Muri L. (2020). Vaccine against peanut allergy based on engineered virus-like particles displaying single major peanut allergens. J. Allergy Clin. Immunol..

[34] Flicker S., Linhart B., Wild C., Wiedermann U., Valenta R. (2013). Passive immunization with allergen-specific IgG antibodies for treatment and prevention of allergy. Immunobiology.

[35] Gevaert P., De Craemer J., De Ruyck N., Rottey S., de Hoon J., Hellings P.W., Volckaert B., Lesneuck K., Orengo J.M., Atanasio A. (2022). Novel antibody cocktail targeting Bet v 1 rapidly and sustainably treats birch allergy symptoms in a phase 1 study. J. Allergy Clin. Immunol..

[36] Dantzer J.A., Wood R.A. (2018). The use of omalizumab in allergen immunotherapy. Clin. Exp. Allergy.

[37] Chinthrajah S., Cao S., Liu C., Lyu S.C., Sindher S.B., Long A., Sampath V., Petroni D., Londei M., Nadeau K.C. (2019). Phase 2a randomized, placebo-controlled study of anti-IL-33 in peanut allergy. JCI Insight.

